# Gene name errors are widespread in the scientific literature

**DOI:** 10.1186/s13059-016-1044-7

**Published:** 2016-08-23

**Authors:** Mark Ziemann, Yotam Eren, Assam El-Osta

**Affiliations:** 1 Baker IDI Heart & Diabetes Institute, The Alfred Medical Research and Education Precinct, Melbourne, Victoria 3004 Australia; 2 Faculty of Engineering, Monash University, Clayton, Victoria 3168 Australia; 3 Central Clinical School, Faculty of Medicine, Monash University, Clayton, Victoria 3168 Australia

**Keywords:** Microsoft Excel, Gene symbol, Supplementary data

## Abstract

The spreadsheet software Microsoft Excel, when used with default settings, is known to convert gene names to dates and floating-point numbers. A programmatic scan of leading genomics journals reveals that approximately one-fifth of papers with supplementary Excel gene lists contain erroneous gene name conversions.

**Electronic supplementary material:**

The online version of this article (doi:10.1186/s13059-016-1044-7) contains supplementary material, which is available to authorized users.

The problem of Excel software (Microsoft Corp., Redmond, WA, USA) inadvertently converting gene symbols to dates and floating-point numbers was originally described in 2004 [1]. For example, gene symbols such as *SEPT2* (Septin 2) and *MARCH1* [Membrane-Associated Ring Finger (C3HC4) 1, E3 Ubiquitin Protein Ligase] are converted by default to ‘2-Sep’ and ‘1-Mar’, respectively. Furthermore, RIKEN identifiers were described to be automatically converted to floating point numbers (i.e. from accession ‘2310009E13’ to ‘2.31E+13’). Since that report, we have uncovered further instances where gene symbols were converted to dates in supplementary data of recently published papers (e.g. ‘*SEPT2*’ converted to ‘2006/09/02’). This suggests that gene name errors continue to be a problem in supplementary files accompanying articles. Inadvertent gene symbol conversion is problematic because these supplementary files are an important resource in the genomics community that are frequently reused. Our aim here is to raise awareness of the problem.

We downloaded and screened supplementary files from 18 journals published between 2005 and 2015 using a suite of shell scripts. Excel files (.xls and.xlsx suffixes) were converted to tabular separated files (tsv) with ssconvert (v1.12.9). Each sheet within the Excel file was converted to a separate tsv file. Each column of data in the tsv file was screened for the presence of gene symbols. If the first 20 rows of a column contained five or more gene symbols, then it was suspected to be a list of gene symbols, and then a regular expression (regex) search of the entire column was applied to identify gene symbol errors. Official gene symbols from Ensembl version 82, accessed November 2015, were obtained for *Arabidopsis thaliana*, *Caenorhabditis elegans*, *Drosophila melanogaster*, *Danio rerio*, *Escherichia coli*, *Gallus gallus*, *Homo sapiens*, *Mus musculus*, *Oryza sativa* and *Saccharomyces cerevisiae* [2]. The regex search used was similar to that described previously by Zeeberg and colleagues [1], with the added screen for dates in other formats (e.g. DD/MM/YY and MM-DD-YY). To expedite analysis of supplementary files from multi-disciplinary journals, we limited the articles screened to those that have the keyword ‘genome’ in the title or abstract (*Science*, *Nature* and *PLoS One*). Excel files (.xls and.xlsx) deposited in NCBI Gene Expression Omnibus (GEO) [3] were also screened in the same way (files released 2005–2015). All URLs screened, results and scripts used in this study are currently available at SourceForge (https://sourceforge.net/projects/genenameerrorsscreen/). Scripts were run on Ubuntu v14.04 LTS with GNU bash, version 4.3.11. These findings were verified manually by downloading and checking Excel files from every paper and GEO file suspected to include gene name errors.

Supplementary files in Excel format from 18 journals published from 2005 to 2015 were programmatically screened for the presence of gene name errors. In total, we screened 35,175 supplementary Excel files, finding 7467 gene lists attached to 3597 published papers. We downloaded and opened each file with putative gene name errors. Ten false-positive cases were identified. We confirmed gene name errors in 987 supplementary files from 704 published articles (Table 1; for individual listings, see Table S1 in Additional file 1). Of the selected journals, the proportion of published articles with Excel files containing gene lists that are affected by gene name errors is 19.6 %. Of the journals selected, *Molecular Biology and Evolution*, *Bioinformatics*, *DNA Research* and *Genome Biology and Evolution* exhibited the lowest proportion (<10 %) of affected papers (Fig. 1a). Journals that had the highest proportion of papers with affected supplementary files were *Nucleic Acids Research*, *Genome Biology*, *Nature Genetics*, *Genome Research*, *Genes and Development* and *Nature* (>20 %). There was a positive correlation between 2015 journal impact factor (JIF) and the proportion of supplementary gene lists affected (Spearman rho = 0.52, two-sided *p* value = 0.03), which might be due to larger and more numerous datasets accompanying high-JIF papers. Of note, *BMC Bioinformatics*, the forum where the Excel gene name issue was originally reported [1], continues to suffer, with gene name errors present in 13.8 % of papers with Excel gene lists. Indeed, the number of papers with gene name errors continues to be a problem (Fig. 1b). Linear-regression estimates show gene name errors in supplementary files have increased at an annual rate of 15 % over the past five years, outpacing the increase in published papers (3.8 % per year). We screened 4321 Excel files deposited to NCBI GEO [3], identifying 574 files with gene lists and finding that 228 (39.7 %) of these contain gene name errors. These are listed in Table S1 in Additional file 1.

**Table 1:** Results of the systematic screen of supplementary Excel files for gene name conversion errors

Journal^a^	Number of Excel files screened	Number of gene lists found	Number of papers with gene lists	Number of supplementary files affected	Number of papers affected	Number of gene names converted
*PLoS One*	7783	2202	994	220	170	4240
*BMC Genomics*	11464	1650	801	218	158	4932
*Genome Res*	2607	580	251	114	68	3180
*Nucleic Acids Res*	2117	540	315	88	67	1661
*Genome Biol*	2678	664	257	97	63	1878
*Genes Dev*	932	395	190	75	55	1593
*Hum Mol Genet*	980	372	168	48	27	1724
*Nature*	482	150	74	27	23	1375
*BMC Bioinformatics*	1790	235	152	26	21	534
*RNA*	569	127	77	20	15	1341
*Nat Genet*	264	70	37	12	9	178
*Bioinformatics*	731	112	67	11	6	339
*PLoS Comput Biol*	177	79	32	6	6	46
*PLoS Biol*	143	54	29	7	5	206
*Mol Biol Evol*	995	112	79	7	4	56
*Science*	172	36	19	7	3	451
*Genome Biol Evol*	490	32	25	2	2	121
*DNA Res*	801	57	30	2	2	6
*Total*	35175	7467	3597	987	704	23861

^a^The 18 journals investigated are ordered by the number of papers affected by gene name conversion errors

**Fig. 1:**
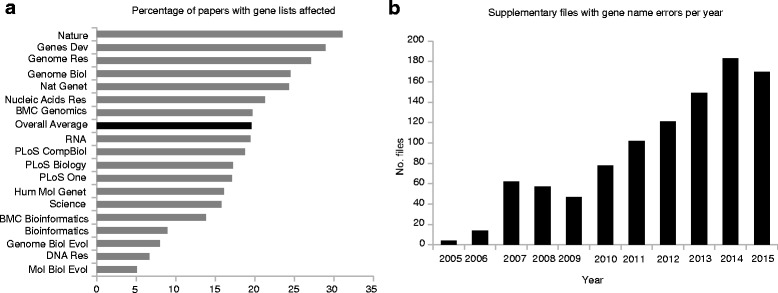
Prevalence of gene name errors in supplementary Excel files. **a** Percentage of published papers with supplementary gene lists in Excel files affected by gene name errors. **b** Increase in gene name errors by year

Automatic conversion of gene symbols to dates and floating-point numbers is a problematic feature of Excel software. The description of this problem and workarounds were first highlighted over a decade ago [1]—nevertheless, we find that these errors continue to pervade supplementary files in the scientific literature. To date, there is no way to permanently deactivate automatic conversion to dates in MS Excel and other spreadsheet software such as LibreOffice Calc or Apache OpenOffice Calc. We note, however, that the spreadsheet program Google Sheets did not convert any gene names to dates or numbers when typed or pasted; notably, when these sheets were later reopened with Excel, LibreOffice Calc or OpenOffice Calc, gene symbols such as *SEPT1* and *MARCH1* were protected from date conversion.

For reviewers and editorial staff, the kind of errors we describe can be spotted by copying the column of gene names and pasting it into a new sheet, and then sorting the column. Any gene symbols converted to dates will appear as numbers at the top of the column. Journals might wish to adapt our supplied scripts to screen for gene name errors in supplementary files or have researchers do this before submission. In the 987 supplementary files containing gene name errors identified here, 166 files did not contain any other identifying information such as accession numbers or genomic coordinates that could be used to infer the original gene names. We recommend that these 166 files be corrected (listed in Table S1 in Additional file 1). We also recorded several cases where gene name errors were located in the first few lines of a file—this suggests to us that these files were not properly reviewed before publication.

Finally, as our scripts focused on screening vertical lists of genes, we might have missed instances of gene symbol errors in horizontal gene lists. There are undoubtedly many more instances of gene name errors in journals outside of the 18 we screened here. In this study, we were not able to programmatically access pay-walled supplementary files. We recommend publishers allow open access to supplementary materials, as exemplified by *Science*, *Nature* and *Nature Genetics*. In conclusion, we show that inadvertent gene name conversion errors persist in the scientific literature, but these should be easy to avoid if researchers, reviewers, editorial staff and database curators remain vigilant.

## Additional file

Additional file 1: Table S1. List of supplementary files containing Excel gene name errors from journals and Gene Expression Omnibus (GEO). (XLSX 81 kb)

## Abbreviations

GEO: Gene Expression Omnibus
JIF: journal impact factor
